# Dual Crosslinked Gelatin Methacryloyl Hydrogels for Photolithography and 3D Printing

**DOI:** 10.3390/gels5030034

**Published:** 2019-07-03

**Authors:** Gozde Basara, Xiaoshan Yue, Pinar Zorlutuna

**Affiliations:** Aerospace and Mechanical Engineering Department, University of Notre Dame, Notre Dame, IN 46556, USA

**Keywords:** dual crosslinking, photocrosslinking, enzymatic crosslinking, microbial transglutaminase, photolithography, 3D printing

## Abstract

Gelatin methacryloyl (GelMA) hydrogels have been used in tissue engineering and regenerative medicine because of their biocompatibility, photopatternability, printability, and tunable mechanical and rheological properties. However, low mechanical strength limits their applications in controlled drug release, non-viral gene therapy, and tissue and disease modeling. In this work, a dual crosslinking method for GelMA is introduced. First, photolithography was used to pattern the gels through the crosslinking of methacrylate incorporated amine groups of GelMA. Second, a microbial transglutaminase (mTGase) solution was introduced in order to enzymatically crosslink the photopatterned gels by initiating a chemical reaction between the glutamine and lysine groups of the GelMA hydrogel. The results showed that dual crosslinking improved the stiffness and rheological properties of the hydrogels without affecting cell viability, when compared to single crosslinking with either ultraviolet (UV) exposure or mTGase treatment. Our results also demonstrate that when treated with mTGase, hydrogels show decreased swelling properties and better preservation of photolithographically patterned shapes. Similar effects were observed when three dimensional (3D) printed and photocrosslinked substrates were treated with mTGase. Such dual crosslinking methods can be used to improve the mechanical properties and pattern fidelity of GelMA gels, as well as dynamic control of the stiffness of tissue engineered constructs.

## 1. Introduction

The extracellular matrix (ECM) is the combination of molecules that provide structure and stability to biological tissues. In the realm of tissue engineering, biologically active scaffolds serve to mimic the native ECM [1,2,3]. ECM properties vary based on their biological functions, so it is crucial to develop scaffolds with adjustable chemical and mechanical properties in order to manipulate cell behavior [4]. Various types of natural and synthetic materials were used to create such scaffolds [5,6,7,8]. Gelatin methacryloyl (GelMA), a photocrosslinkable hydrogel obtained by modifying gelatin, is one of the most attractive materials due to its biocompatibility, tunable rheological and mechanical properties, low cost and printability [9,10]. Moreover, photopatterning can be used to fabricate micropatterned GelMA scaffolds [9,11,12]. However, its relatively poor mechanical properties, especially in the low concentrations frequently used for better cell viability [9,13,14,15] as well as improved cell spreading and migration [9], can be a limitation for tissue engineering applications where high ECM stiffness is required [16].

Existing strategies to improve the mechanical properties of GelMA gels usually involve the introduction of other materials. Biopolymers such as dextran [17], silk fibroin [18], hyaluronic acid, [19] and gellan gum [20] have been incorporated into GelMA gels to tune their mechanical properties. In addition, reinforcement with 3D printed carbon nano-tubes [21,22], microfibers [16], polyacrylamide [23], graphene oxide [24], polyethylene glycol [25,26] and polylactide-co-ethylene oxide-co-fumarate (PLEOF) have been reported to alter the physical properties of GelMA gels. Introducing another material into GelMA hydrogels could require tedious processes, and more importantly cannot be done in a dynamic fashion. For example, to study the myocardial infarction in vitro, dynamic stiffening of the engineered hydrogels introduced by enzymatic crosslinking will be valuable, since it is shown that the tissue becomes stiffer after myocardial infarction [27]. This feature can also be used to study the effect of gradual stiffening of the substrate on breast cancer cells, as it is shown that the tissue ECM often stiffens when cancer occurs [28].

Alternative methods have been explored to tune the mechanical properties of GelMA using multiple crosslinking steps. For example, Rizwan et. al. used a sequential crosslinking approach in which a physical crosslinking step was performed by placing the gels at 4 °C prior to the photocrosslinking step [29]. In another study, Zhou et. al. used enzymatic crosslinking followed by photocrosslinking in order to improve the viscosity of GelMA for bioprinting purposes [30]. They mixed mTGase powder with GelMA and studied the viscoelastic properties of the constructed gels, resulting in better shear thinning properties and improved printability in extrusion-based printing [31]. Both studies demonstrated improved rheological properties of GelMA while enabling the fabrication of the hydrogels in predesigned shapes.

In this study, we introduced a different two-step crosslinking method, photocrosslinking followed by enzymatic crosslinking, in order to form dynamically tunable GelMA hydrogels with improved mechanical properties. First, photocrosslinking was used to construct the initial shape of the hydrogel. Then, using Ca^2+^ independent mTGase solution, the hydrogel was further crosslinked enzymatically, resulting in up to a seven-fold improvement in mechanical strength. The fabricated GelMA hydrogels were characterized in terms of compressive Young’s modulus, rheology, swelling, degradation, and cell viability. Dual crosslinking strategies that are biocompatible and non-cytotoxic can potentially be used at any moment during the culture of the tissue engineered constructs. The ability to dynamically change the stiffness of the substrate would be beneficial in disease modeling applications where the stiffness of the tissue changes due to the disease pathology.

## 2. Results and Discussion

### 2.1. Methacrylation of Gelatin

**Figure 1:**
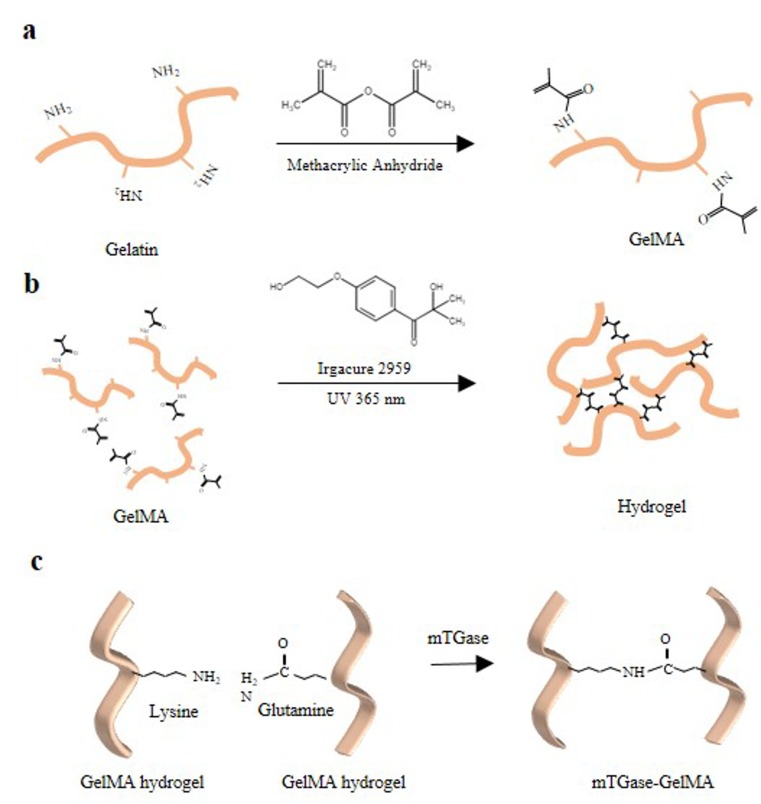
Schematic elucidating the crosslinking mechanism of GelMA (**a**) Modification of gelatin to GelMA (**b**) Photocrosslinking of GelMA hydrogel (**c**) Enzymatic crosslinking of GelMA hydrogel.

GelMA was synthesized by incorporating methacrylate groups onto the amine containing side groups of gelatin (Figure 1a). The degree of methacrylation (DM) can be quantified by the percentage of the lysine groups that are substituted by the methacrylate groups, which was set to 52.5% ± 0.97% in this study. The non-modified groups in GelMA serve as the sites for further enzymatic crosslinking by mTGase, which initiates a chemical reaction between glutamine and lysine groups within GelMA hydrogel (Figure 1c). This reaction improves the strength of the 3D hydrogels by providing extra support from the amide bonds. 

### 2.2. Rheological Characterization

**Figure 2:**
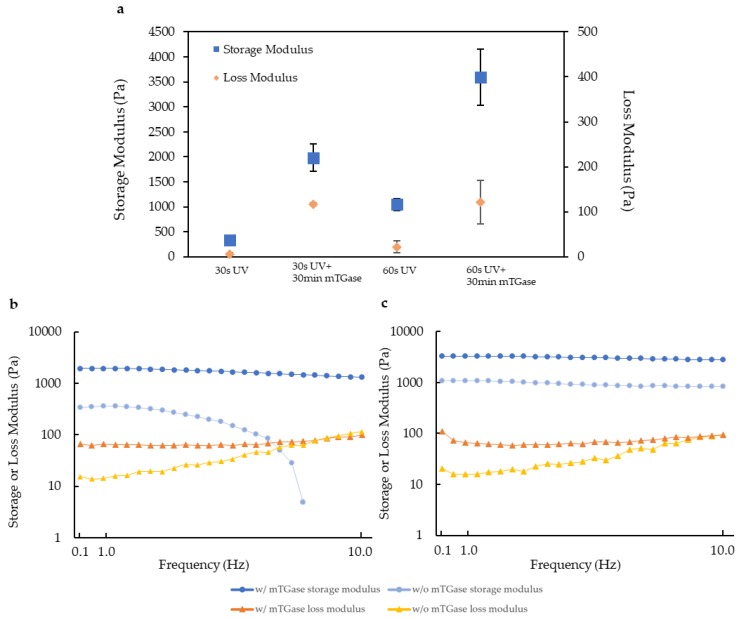
Viscoelastic properties of hydrogels crosslinked with 30 s and 60 s of UV exposure with or without mTGase treatment (**a**) time sweep results (**b**) frequency sweep results for 30 s of UV exposure condition (**c**) frequency sweep results for 60 s of UV exposure condition. (w/: with; w/o: without; mTGase: microbial transglutaminase).

To investigate the viscoelastic properties of the dual crosslinked gels, rheological characterization was performed using time sweep and frequency sweep tests. In the time sweep test, the storage and the loss modulus were recorded for 1 min and were found to be almost constant without mTGase treatment. With mTGase treatment, there was an increase in both the storage and the loss modulus for both 30 s and 60 s of UV photocrosslinking conditions (Figure 2a). The frequency sweep test showed a decrease in the storage modulus, whereas the loss modulus increased with the increasing frequency for both 30 s and 60 s of UV crosslinking conditions and with or without the mTGase treatment. Regardless of UV exposure time, the mTGase treatment increased the storage and the loss modulus (Figure 2b). For the lower frequency values, the storage modulus was greater than the loss modulus, which indicates predominantly elastic behavior rather than viscous behavior [32]. Our results showed that for 30 s of UV crosslinking condition, the gel structure was lost for single crosslinked gels, whereas mTGase treated gels retained their structure. Moreover, storage modulus indicates the ability to store deformation energy in an elastic manner and increases with the degree of crosslinking. Our results confirmed that the mTGase treatment increased the extent of crosslinking and enhanced the viscoelastic properties of the hydrogels.

### 2.3. Swelling and Mechanical Characterization

**Figure 3:**
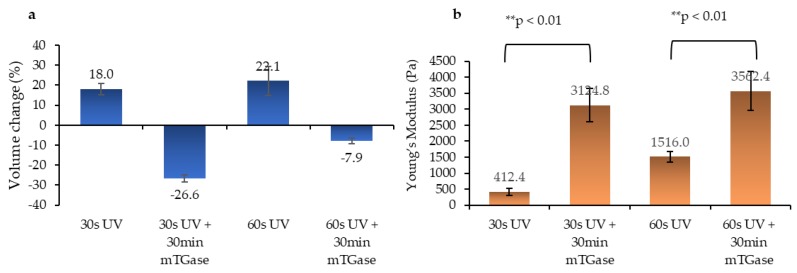
Swelling and mechanical characteristics of the gels (**a**) Swelling properties (**b**) Stiffness analysis using nanoindentation.

The volume of the hydrogels was measured 1 day after incubation in phosphate-buffered saline (PBS). Photocrosslinking alone, with 30 and 60 s of UV exposure, resulted in hydrogels that were swollen by 18% (± 3%) and 22.1% (± 7%) of their initial volume, respectively. Thus, no significant difference was observed between the two exposure times. On the contrary, when hydrogels were further crosslinked with mTGase for 30 min right after UV crosslinking (dual crosslinking), they shrunk slightly, instead of swelling (Figure 3a). This is likely a result of increased crosslink levels in the dual crosslinked hydrogels, which led to higher rigidity and lower water content. In parallel with the literature, the Young’s modulus of the gels increased with increased UV treatment time [33]. Furthermore, stiffness test results showed that the treatment with mTGase increased hydrogel stiffness seven-fold for 30 s of UV crosslinking conditions and 2-fold for 60 s of UV crosslinking conditions (Figure 3b), while no significant difference between the Young’s modulus of mTGase treated samples when photocrosslinked with 30 s or 60 s of UV exposure was observed. Dual crosslinking was less effective at increasing hydrogel stiffness after 60 s of UV exposure than after 30 s. This might have been caused by the competition between the two crosslinking strategies, both of which utilize lysine groups for crosslinking to occur. Treatment with mTGase causes covalent bonding between adjacent polymers, which improves the internal structure of the gel and results in improved stiffness. As UV treatment time increases, the number of crosslinked groups occupied with methacrylic anhydride (MAnh) increases, therefore the potential for chemical crosslinking decreases. 

### 2.4. Degradation Test

To observe the effect of mTGase treatment on hydrogel degradation, collagenase digestion analysis of the single crosslinked (photocrosslinked with 30 s of UV exposure time) and dual crosslinked (photocrosslinking with 30 s of UV exposure time followed by 30 min mTGase treatment) gels were performed in vitro.

**Figure 4:**
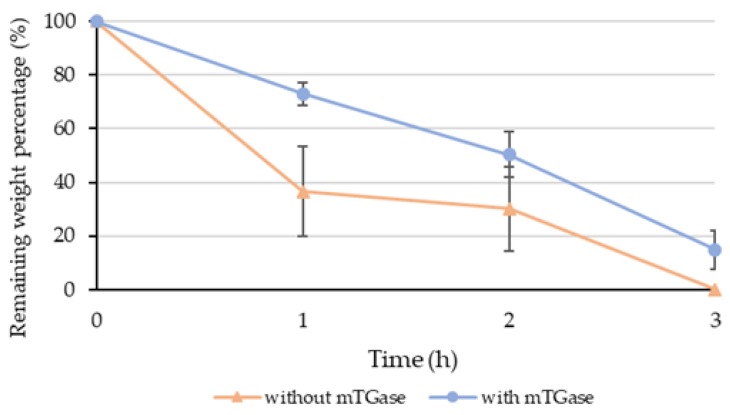
Enzymatic degradation of GelMA gels with or without mTGase treatment (mTGase: microbial transglutaminase).

It was observed that after an hour, only 36.6% of the UV crosslinked gels remained, whereas 73.0% of dual crosslinked gels remained. After 3 h, single crosslinked gels completely degraded, whereas 14.8% of the dual crosslinked hydrogels still remained (Figure 4). These results are consistent with the literature where it has been reported that UV crosslinked GelMA gels degrade faster compared to GelMA gels crosslinked using thermal crosslinking followed by photocrosslinking [29].

### 2.5. 3D Printing

Using 3D printing, patterning can be done for more complex shapes in larger dimensions. However, the UV crosslinking method usually requires a long exposure time or high UV intensity to obtain mechanically manageable hydrogel structures, which largely inhibits hydrogel 3D printing in combination with cells for biological applications. To solve this problem, we further tested the dual crosslinking strategy for reinforcing the 3D printed structures.

**Figure 5:**
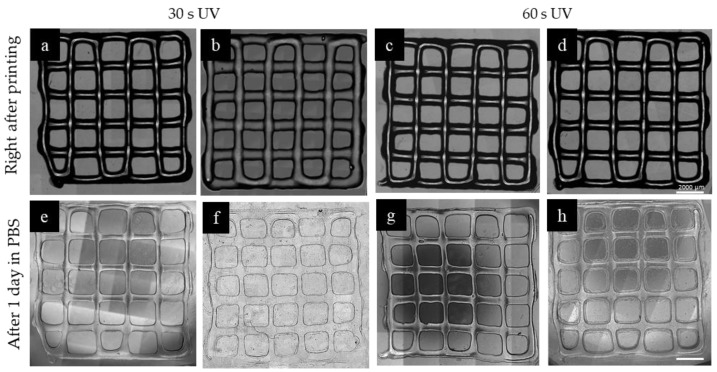
3D printed constructs made of 2 layers, prepared using (**a**,**b**,**e**) 30 s of UV crosslinking without mTGase treatment, (**f**) 30 s of UV crosslinking with 30 min mTGase treatment, (**c**,**d**,**g**) 60 s of UV crosslinking without mTGase treatment, (**h**) 60 s of UV crosslinking with 30 min mTGase treatment. The treatment with mTGase reduced the swelling and helped the structure to preserve its shape. (Scale shows 2000 µm)

**Figure 6:**
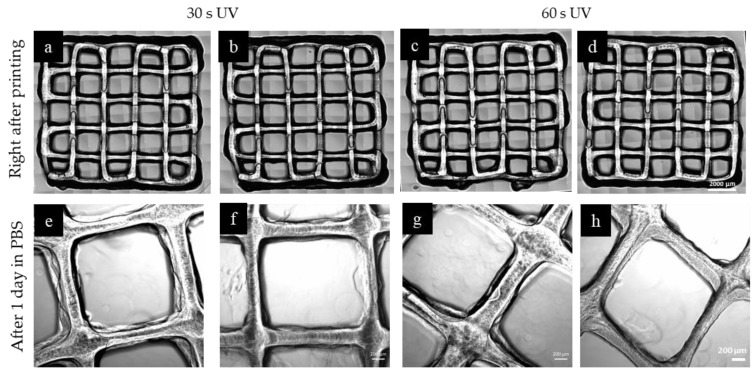
3D printed constructs made of 6 layers, prepared using (**a**,**b**,**e**) 30 s of UV crosslinking without mTGase treatment, (**f**) 30 s of UV crosslinking with 30 min mTGase treatment, (**c**,**d**,**g**) 60 of s UV crosslinking without mTGase treatment, (**h**) 60 s of UV crosslinking with 30 min mTGase treatment. (Scale shows 2000 µm for upper row and 200 µm for lower row)

To investigate the effect of mTGase treatment on 3D printed samples as a proof of concept, grid-shaped constructs consisting of two layers were printed. The printed shape is a square (10 mm × 10 mm), and the distance between the two neighboring lines is 2 mm. **In all samples, it was observed that the corners of the squares were not as sharp and showed a slight discrepancy from the 3D model, which is expected when printing with hydrogel materials. The brightfield images of the samples were taken immediately after printing. The samples were divided into three groups. The first group was only photocrosslinked with 30 s and 60 s of UV exposure. The second group was only enzymatically crosslinked by using mTGase solution. Immediately after printing, the constructs were soaked in mTGase solution and kept in an incubator for 30 min. The final group was dual crosslinked. For the last group, upon completion of the entire structure, photocrosslinking was followed by enzymatic crosslinking. The results showed that the group which was only enzymatically crosslinked with mTGase solution did not crosslink properly, and the gel structure collapsed right after adding PBS. Similarly, in literature, it was reported that when the substrates were printed by using gelatin-only bioink and soaked in mTGase solution, greater disintegration occurred compared to the ones printed with the bioink containing gelatin and mTGase [34]. The remaining sample groups were kept in PBS solution at 37 °C overnight before taking the next set of images. Figure 5 shows the brightfield images of each group. Both treated and non-treated samples preserved their shapes and resolution. To investigate the effect of sample height, the same pattern was printed with six layers by following the previously described procedure. The results are shown in Figure 6. Brightfield images showed that, immediately after printing, all the patterns were similar. Also, the pore volume was preserved during printing, which is an indication of successful printing [35]. However, after one day in PBS, the patterns preserved their resolution better in the mTGase treated cases for both 30 s and 60 s of UV treatment time conditions, **which was verified by measuring the distance between neighboring lines on the second day. This distance was measured as 1.6 mm for the dual crosslinked sample and 1.4 mm for the sample that was photocrosslinked only. This difference in accuracy is likely due to the swelling of the hydrogel material, and thus the mTGase treatment enhanced the accuracy of the printing due to the lower degree of swelling of the dual crosslinked hydrogels. 

### 2.6. Cell Viability

**Figure 7:**
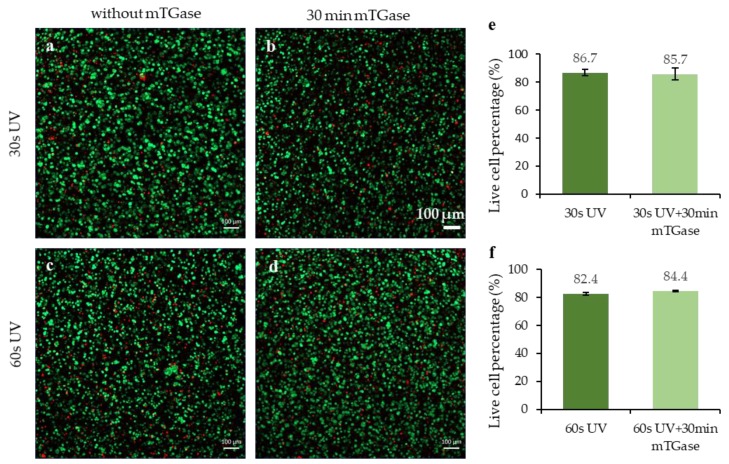
Cell viability analysis of cell-laden micropatterned gels prepared using (**a**) 30 s of UV treatment without mTGase treatment (**b**) 30 s of UV treatment with 30 min mTGase treatment, (**c**) 60 s of UV treatment without mTGase treatment, (**d**) 60 s of UV treatment with 30 min mTGase treatment, (**e**) live cell percentage comparison for 30 s of UV treatment with or without mTGase treatment and (**f**) live cell percentage comparison for 60 s of UV treatment with or without mTGase treatment.

To observe the effect of dual crosslinking on cell viability, human breast cancer cell line HCC1806 cells were encapsulated in GelMA, and live/dead staining was performed on the next day. The results for only photocrosslinked and dual crosslinked conditions were compared. For each condition, 3 samples (*n* = 3) were stained and the average was taken. No significant difference was observed between the dual crosslinked and photocrosslinked conditions for both 30 s and 60 s of UV exposure duration (Figure 7a–d). For 30 s of UV exposure, the percentages of live cells were calculated as 86.7% ± 2.1% and 85.7% ± 4.1% for photocrosslinked and dual crosslinked conditions, respectively (Figure 7e). For 60 s of UV exposure, the percentages of live cells were calculated as 82.4% ± 1.1% and 84.4% ± 0.4% for photocrosslinked and dual crosslinked conditions, respectively (Figure 7f). This result indicates that mTGase is not toxic to the cells and hence can be used for further crosslinking cell-laden gels, which is in agreement with the literature [36].

**Figure 8:**
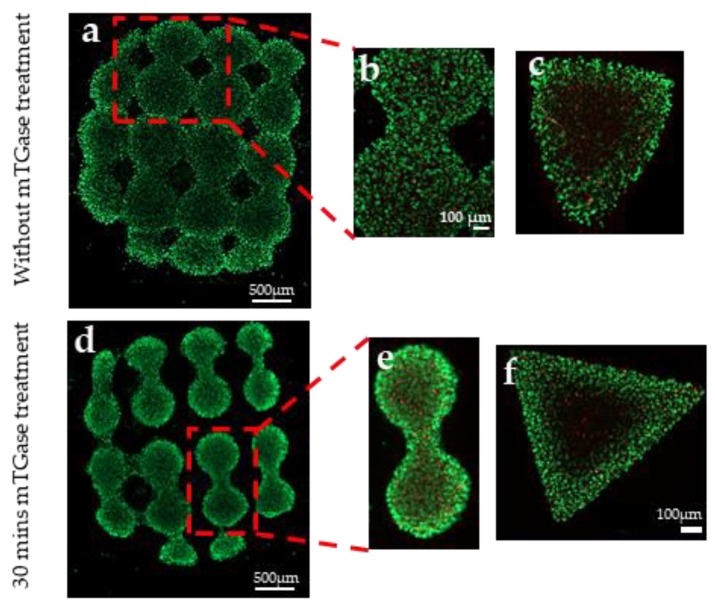
Micropatterned cell-laden gels created using photolithography (**a**) dumbbell patterns, (**b**) zoomed in view of dumbbell pattern, (**c**) triangular pattern, (**d**) dumbbell patterns, (**e**) zoomed in view of dumbbell pattern, (**f**) triangular pattern.

To study the pattern fidelity of photocrosslinking using the dual crosslinking method we describe here, dumbbell and triangle shaped masks were used during UV exposure. As a result, cells were encapsulated as micropatterned, cell-laden gels. To clearly visualize the patterns, live/dead staining was performed one day after encapsulation and results are presented in Figure 8. The figure demonstrates that hydrogel patterns were successfully fabricated. In the absence of the mTGase treatment, the pattern showed a higher swelling ratio, and the shape was disturbed. We quantified the swelling by measuring the largest distance between the patterns, which was approximately 120 µm greater for treated samples. It is well-known that an increase in crosslinking density results in a reduced swelling, and our results are in agreement with that [37].

## 3. Conclusions

The dual crosslinking method presented in this study can be used to improve the mechanical and rheological properties of the gelatin methacryloyl hydrogels in a cytocompatible manner. The first step, which entails photocrosslinking, can be used to create micropatterned cell-laden hydrogels. Then, by introducing mTGase, further crosslinking can be achieved to improve the mechanical properties and to increase the pattern fidelity by controlling the excessive swelling. Our results confirmed that the Young’s modulus and the storage modulus of the gels improved significantly with mTGase treatment. Additionally, photopatterned as well as 3D printed gels retained their shapes better without affecting cell viability. 

The second step of the presented dual crosslinking method can be applied at any time after initial photocrosslinking, and therefore can be used to mimic the dynamic stiffness changes which are common in many pathophysiological conditions such as cancer and heart attack. 

## 4. Materials and Methods

### 4.1. Materials

MAnh, photo initiator (PI) Irgacure 2959, gelatin (gel strength 300g Bloom, Type A, from porcine skin) was purchased from Sigma-Aldrich (St. Louis, MO, USA). 12–14 kDa dialysis tube and Trypsin ethylenediamine tetraacetic acid (EDTA) were purchased from VWR (Chicago, IL, USA). Deuterium oxide (D_2_O) was purchased from Cambridge Isotope Laboratories Inc (Andover, MA, USA). DMEM High Glucose and PBS were purchased from Corning (Corning, NY, USA). Fetal bovine serum (FBS) was purchased from Hyclone (South Logan, UT, USA) and penicillin-streptomycin (P/S) was purchased from Gibco (Waltham, MA, USA). Live/dead assay was purchased from Life Technologies (Carlsbad, CA, USA). The mTGase was a kind gift from Ajinomoto (Sunburg, MN, USA) and it has an enzymatic activity of 100 U/g. 

### 4.2. Synthesize of GelMA

GelMA was synthesized by following the previously established protocol [38]. Briefly, 10 g of gelatin was dissolved in 100 mL of PBS at 60 °C. After completely dissolving, 2mL of MAnh was added dropwise and the solution was kept at 60 °C and left to react 3 h. After 3 h, 400 mL of PBS, pre-warmed to 40–50 °C, was added to the solution and mixed for 15 min. The solution was then transferred into a dialysis tube and dialyzed against deionized (DI) water at 40–50 °C with constant stirring. The dialysis was run for one week, with twice daily water changes. Finally, the GelMA solutions were filtered and lyophilized for further use. 

### 4.3. Preparation and Crosslinking of Hydrogels

The hydrogel solution was prepared by dissolving GelMA (5% *w*/*v*) and adding PI (0.1% *w/v* in PBS). To dissolve GelMA in PBS, the mixture was kept in a water bath at 37 °C for 5 min. The solution was then transferred onto a stage with 100 µm thick spacers and sandwiched between the stage and a glass slide. The gels were then exposed to 6.9 mW/cm^2^ UV irradiation by using a UV lamp (Lumen Dynamics, (Mississauga, ON, Canada). As a result, circular shaped gels were obtained. The hydrogels were prepared at room temperature, and immediately placed in 37 °C.

The mTGase solution was prepared with DI water (80 mg/mL *w*/*v*). The solution was mixed thoroughly using a vortex and kept at 37 °C to provide complete dissolution of mTGase. The gels were prepared using the conditions shown in Table 1. To determine the effect of treatment time, two different time points were tried: the gels were soaked in mTGase solution for 30 min and overnight. It was observed that 30 min treatment improved the mechanical properties, whereas the overnight application decreased them. Further optimization can be performed in future studies to find the best enzyme treatment conditions.

### 4.4. Materials Characterization

#### 4.4.1. ^1^H NMR Characterization

The methacrylation of gelatin was characterized with an ^1^H NMR spectroscopy. GelMA solution was prepared with a concentration of 30 mg/mL in D_2_O and ^1^H NMR spectra were collected. After baseline correction, the areas of the peaks were integrated. The degree of methacrylation, which is the percentage of ε-amino groups of gelatin modified with MAnh, was calculated by using Equation (1).

(1) $$\mathrm{DM}\left(\%\right)=\;1-\frac{\mathrm{GelMA}\;\mathrm{lysine}\;\mathrm{methylene}\;\mathrm{area}\;}{\mathrm{Gelatin}\;\mathrm{lysine}\;\mathrm{metylene}\;\mathrm{area}}$$

#### 4.4.2. Rheological Properties

Two different rheological tests were conducted to characterize the viscoelastic properties of the only photocrosslinked and dual crosslinked gels. For both experiments, 8 mm diameter parallel-plate geometry was used with a crosshatch plate. A time sweep test was done to study the strength of the structure of the gels. For this test, storage modulus (G’) and loss modulus (G’’) were recorded as a function of time under a fixed frequency of 1 Hz and strain of 3%. Each gel was tested at 37 °C for 1 min. During testing, measurements were taken every 10 s and the average of six data points shows the average modulus of each gel.

The frequency sweep test was done to observe the viscoelastic properties of the gels. The storage modulus and loss modulus were recorded over a frequency range from 0.1 to 10 Hz with 2% strain at 37 °C.

#### 4.4.3. Mechanical Properties

To determine the mechanical properties, compression stiffness test was conducted by using a nanoindenter (Optics 11, Westwood, MA, USA) with an indentation probe (spring constant of 0.45 N/m, tip diameter of 50µm). Young’s modulus of each hydrogel was calculated using in house MATLAB code, which determines the slope of the stress-strain plot in the elastic region.

#### 4.4.4. Swelling Test

The swelling properties of hydrogels were measured by observing the volume changes between the hydrogels that were freshly prepared and those that were incubated in PBS solution for 24 h. The volumes of the gels were calculated by multiplying the surface area with the thickness. To calculate the surface area, images of the gels (*n* = 3) were taken immediately after preparation and on the next day and measured with ImageJ (version 1.51k, National Institutes of Health Bethesda, MD, USA). The thickness of the gels was 1 mm right after preparation, which was decided by the height of the stage used for hydrogel fabrication. One day after fabrication, the thickness of the gels was determined by the force reading in the rheometer. The upper head of the rheometer was lowered until it touched the surface of the gel. At the contact point, the force reading started increasing. The point when the force reading started increasing from zero was used as the contact point to decide the thickness of each gel.

#### 4.4.5. Degradation Test

For the degradation test, the hydrogel solution was prepared by dissolving GelMA (5% *w*/*v*) and adding PI (0.1% *w/v* in PBS). From this solution, 30 µl disks were prepared using PDMS molds 6 mm in diameter and 1 mm thick, then crosslinked with either only 30 s UV exposure or 30 s UV exposure followed by 30 min mTGase (80 mg/mL *w/v* in DMEM) treatment. After polymerization, the hydrogel discs were transferred to 24-well plates and allowed to swell in DMEM medium supplemented with FBS and penicillin in an incubator at 37 °C overnight. The next day, the medium was removed and 1 mL of collagenase type 2 (240 U/mg, Worthington Biochemical, Lakewood, NJ, USA) with 5 U/mL concentration in DMEM was added to each well. The weight of the hydrogels was measured after carefully drying with a delicate tissue wipe (KimTech Science, Roswell, GA, Canada), every hour for 3 h. For each time point, 3 samples were used, and the remaining weight (RW) percentage was calculated using Equation (2).

(2) $$\mathrm{RW}\;\left(\%\right)=\;100+\frac{\left(\mathrm{weight}\;\mathrm{at}\;\mathrm{required}\;\mathrm{time}\;\mathrm{point}-\mathrm{initial}\;\mathrm{weight}\right)}{\mathrm{initial}\;\mathrm{weight}}*100$$

#### 4.4.6. 3D Printing

For 3D printing, bioink was prepared by dissolving GelMA (10% *w*/*v*) in PBS and adding PI (0.1% *w/v* in PBS). The bioink was then transferred into the cartridge while it was still liquid. The cartridge was put in 4 °C for 5 min to achieve the required viscosity prior to printing. A CELLINK Inkredible+ Bioprinter was used for printing. Printing was done at room temperature, with a pressure range of 50–57 kPa. The translational speed of the nozzle was 75 mm/min and the nozzle diameter was 250 µm. By using a custom-made G-code, a grid pattern consisting of two layers with dimensions of 1 cm × 1 cm was printed into each well of a 12-well plate. Printed constructs were either treated with 30 s or 60 s UV only, or they went through mTGase treatment for 30 min following the UV crosslinking. All samples were kept at 37 °C in PBS solution overnight. Brightfield images were taken right after fabrication as well as 24 h after incubation.

For 3D printing, bioink was prepared by dissolving GelMA (10% *w*/*v*) in PBS and adding PI (0.1% *w/v* in PBS). The bioink was then transferred into the cartridge while it was still liquid. The cartridge was put in 4 °C for 5 min to achieve the required viscosity prior to printing. A CELLINK Inkredible+ Bioprinter was used for printing. Printing was done at room temperature, with a pressure range of 50–57 kPa. The translational speed of the nozzle was 75 mm/min and the nozzle diameter was 250 µm. By using a custom-made G-code, a grid pattern consisting of two layers with dimensions of 1 cm × 1 cm was printed into each well of a 12-well plate. Printed constructs were either treated with 30 s or 60 s UV only, or they went through mTGase treatment for 30 min following the UV crosslinking. All samples were kept at 37 °C in PBS solution overnight. Brightfield images were taken right after fabrication as well as 24 h after incubation.

### 4.5. Cell encapsulation and Imaging

#### 4.5.1. Cell Encapsulation for Biocompatibility

Human breast cancer cell line HCC1806 cells were cultured in DMEM high glucose supplemented with 10% FBS and 1% P/S at 37 °C with 5% CO_2_. Cells were collected when they reached approximately 80% confluency and encapsulated in GelMA hydrogels (*n* = 3) with a cell density of 0.1 million cells per construct (5µL total volume). The hydrogel solution was prepared by dissolving GelMA (5% *w*/*v*) in PBS and mixing in PI to achieve a final concentration of 0.1% *w*/*v*. Then, the solution was mixed with cell suspension at a ratio of 1:1 (GelMA:cell solution). The mixture was then transferred onto a stage with 100 µm thick spacers and sandwiched between the stage and a glass slide. The whole structure was exposed to 6.9 mW/cm^2^ UV irradiation for 30 or 60 s. The amount of PI and the dose of UV exposure have been shown to be harmless for cell encapsulation studies [38]. The hydrogels were washed thoroughly with PBS to remove the excess PI. The mTGase solution was prepared with culture media (80 mg/mL *w*/*v*). The solution was mixed thoroughly using a vortex and kept at 37 °C to provide complete dissolution of mTGase. After washing, half of the samples were kept in mTGase solution at 37 °C for 30 min. After 30 min the mTGase solution was replaced with fresh culture media. 

To determine the cell viability, cells were stained with Live/Dead Assay (Life technologies) 24 h after encapsulation, following the manufacturer’s instructions. Live cells were stained with Calcein AM (green), and dead cells were stained with Ethidium homodimer-1 (red). The images were taken with a fluorescence microscope (Zeiss, Hamamatsu ORCA flash 4.0, Thornwood, NY, USA). For each hydrogel, z-serial images were taken at three different locations with optical sectioning, and the background signals were eliminated with structural illumination (Apotome, Zeiss, Thornwood, NY, USA). Live and dead cells were counted in ImageJ software. Live cell percentage was calculated by using Equation (3).

Live cell (%) = [(live cell number)/(total cell number)]*100(3)

#### 4.5.2. Cell Encapsulation for Photolithography

For photolithography test, gels were prepared by using the same GelMA and PI concentrations as described above and mixed with 20 million cells/mL. The mixture was then transferred onto a stage with 100 µm thick spacers and sandwiched between the stage and a glass slide. A photomask with the desired micropatterns was placed on top of the glass slide and exposed to 6.9 mW/cm^2^ UV irradiation for 60 s by using a UV lamp (Lumen Dynamics, Mississauga, ON, Canada).

## Figures and Tables

**Table 1:** Parameters used for gel preparation.

UV Treatment Time (s)	mTGase Treatment Time (min)
30	-
30	30
60	-
60	30
